# Brain ethanolamine phospholipids, neuropathology and cognition: A comparative post-mortem analysis of structurally specific plasmalogen and phosphatidyl species

**DOI:** 10.3389/fcell.2022.866156

**Published:** 2022-08-24

**Authors:** Dayan B. Goodenowe, Vijitha Senanayake

**Affiliations:** Prodrome Sciences USA LLC, Temecula, CA, United States

**Keywords:** plasmalogen, amyloid, neurofibrillary tangle, flotillin, docosahexaenic acid, cognition, Alzheimer’s, brain

## Abstract

Reduced cognition in the elderly is associated with low levels of plasmalogens and high levels of lipid rafts, amyloid plaques, and neurofibrillary tangles in the temporal cortex. A systematic integrative analysis of key indices of these pathologies to determine their collective and independent contributions to cognition was performed. Levels of four phosphatidylethanolamines (PE) and four ethanolamine plasmalogens (PL) of identical sn-1 carbon length and desaturation (stearic, 18:0) and identical sn-2 fatty acid compositions of varying side chain lengths and degrees of unsaturation (oleic, 18:1; linoleic, 18:2; arachidonic, 20:4; docosahexaenoic, 22:6), flotillin-1 expression and amyloid plaque and neurofibrillary tangle densities were measured in inferior temporal cortex tissue from 100 elderly subjects (Rush University Memory and Aging Project, 88.5 ± 5.8 years old). Subjects were evenly distributed with respect to gender (52/48, F/M) and cognitive status (38/24/38, no cognitive impairment/mild cognitive impairment/Alzheimer’s dementia) proximate to death. Multivariate logistic regression analyses were used to determine the relative and collective associations of the neuropathological indices with cognition. Higher levels of tangles, amyloid, or flotillin and lower levels of PL 18:0/22:6 were significantly associated with lower cognition in the base model (adjusted for age, sex, education). Multivariate analysis revealed that only PL 18:0/22:6 (β = 0.506; *p* < 0.00001), tangles (−0.307; *p* < 0.01), and flotillin (−0.2027; *p* < 0.05) were independently associated with reduced cognition. PL 18:0/22:6 and PE 18:0/22:6 levels were independently associated with cognition in the presence of tangles, amyloid, and flotillin, but only PL 18:0/22:6 retained its association with cognition when both PL and PE 18:0/22:6 were included in the model indicating that PE 18:0/22:6 levels were associated with PL 18:0/22:6, not cognition. Only high brain levels of PL 18:0/22:6 (>mean+1SD) was predictive of normal cognition (coef = 1.67, *p* < 0.05) and non-demented state (coef = −2.73, *p* < 0.001), whereas low levels of PL 18:0/22:6 and high levels of tangles or flotillin were predictive of dementia. The association of high brain polyunsaturated (PUFA)-PL levels with better cognition was independent of amyloid plaque, neurofibrillary tangle, PE, and flotillin-1 expression. Maintenance or augmentation of brain docosahexaenoic (DHA)-PL levels warrants further investigation as a target for preventing cognitive decline or improving cognition in the elderly, respectively.

## Introduction

Neurodegeneration leading to dementia and death is emerging as the most important age-related disease of our time. The average time to death from a diagnosis of dementia is only 4.5 years (Xie et al., 2008) and Alzheimer’s disease (AD) is the predominant form of dementia in the elderly. The economic and health cost of dementia is enormous and growing due to an increasingly aged population (Alzheimer’s Association, 2018). Dementia in the elderly is a neuropathological disease. Amyloid plaques and neurofibrillary tangles are pathological hallmarks of AD and were once thought to be not just biomarkers of neuropathological decline, but causative of neuropathological and functional decline in the elderly (Hardy and Selkoe, 2002). However, recent clinical trials have cast doubt on the putative causative roles of these well-established neuropathological markers on cognitive decline (Kozin et al., 2018). The presence of high amyloid plaque densities in cognitively normal persons illustrates that there must be other neuropathological features that modulate the associations of these historical pathologies with cognition (Negash et al., 2011) and which are more proximate to the biochemical mechanisms that are required for normal cognitive function.

Changes in neuronal membrane composition are directly implicated as potential causative mechanisms involved in the pathological accumulation of amyloid (Nitsch et al., 1992) and reduced neuronal synaptic activity (Wurtman, 1992). A higher density of cholesterol-rich lipid raft regions are associated with increased β-secretase activity and increased Aβ_1-42_ production (Kojro et al., 2001; Cordy et al., 2003) and a higher density of polyunsaturated plasmalogen rich regions is associated with increased α-secretase activity and decreased Aβ_1-42_ production (Wood et al., 2011a). Membrane fusion dynamics, the biophysical process through which neurotransmitters are released into the synapse, is dependent upon membrane levels of polyunsaturated fatty acid (PUFA) containing ethanolamine plasmalogens (PL) (Glaser and Gross, 1995). PL is emerging as both a diagnostic and therapeutic target for neuropathological decline and dementia (Goodenowe et al., 2007). Brain PL levels are lower in AD than age-matched controls (Ginsberg et al., 1995; Han et al., 2001; Wood et al., 2011a; Igarashi et al., 2011; Otoki et al., 2021) and low brain levels correlate with low serum levels (Wood et al., 2011a). Serum PL levels correlate with cognition in subjects diagnosed with probable AD, in AD subjects at diagnosis who were later confirmed by autopsy, and in serum samples collected at time of death in subjects with post-mortem AD pathology (Goodenowe et al., 2007). Reductions in metabolic pathways involved in PL biosynthesis are associated with lower cognition (Astarita et al., 2010). Relation of PL and other phospholipid species in the brain in individuals with different cognitive categories (Clinical Dementia Rating) has been previously studied (Han et al., 2001).

Studying the effects of plasmalogen supplementation is challenging due to the complex distribution of molecular species in mammalian tissues (Han et al., 2001) which renders measuring the effects of a single type of plasmalogen difficult using animal extracts. Their extensive gut metabolism and acid degradation during oral administration (Fallatah et al., 2020) creates additional scientific challenges. To overcome these issues, 1-O-alkyl-2-acylglycerols were developed as metabolic precursors to plasmalogens. These precursors can be synthetized to contain specific sn-1 alcohols and specific sn-2 fatty acid side chains. They are stable, orally bioavailable, and dose-dependently elevate PL levels *in vitro* and *in vivo* corresponding to the sn-1 fatty alcohol and sn-2 fatty acid of the precursor (Mankidy et al., 2010; Wood et al., 2011a; Wood et al., 2011b). These precursors enable researchers to precisely assess the effects of plasmalogen supplementation. These plasmalogen precursors are neuroprotective in animal models of Parkinson’s (Miville-Godbout et al., 2016) and multiple sclerosis (Wood et al., 2011a) and improve neurological function in neurologically compromised animals (Gregoire et al., 2015).

The extent to which PL interact with and modulate the association of pathology with cognition in the human brain are not known. We therefore sought to investigate in greater detail the associations between cognition, canonical neuropathology and a series of structurally specific membrane phospholipids known to be altered in persons with AD pathology or reduced cognition. All neurological functions are dependent upon the presynaptic release of neurotransmitters and the subsequent postsynaptic interaction of these neurotransmitters on postsynaptic receivers/receptors. Cognition is dependent upon cholinergic neuron integrity and function (Blusztajn and Wurtman, 1983; Wurtman et al., 1985; Wurtman, 1992). Presynaptic cholinergic axon terminals are unique among neurons in that both the release of its neurotransmitter, acetylcholine, and the reabsorption of the neurotransmitter precursor, choline, is dependent upon membrane fusion due to the unique cellular location of the re-uptake protein. The choline high affinity transporter (CHT) is not constituently expressed on the presynaptic membrane like the neurotransmitter uptake proteins of other neuron types, but instead is expressed only on the presynaptic vesicles and only transiently expressed on the presynaptic membrane during neurotransmitter release (Ferguson et al., 2003). Pharmacological inhibition of CHT with hemicholinium-3 reduces acetylcholine release (Maire and Wurtman, 1985) and choline starvation causes membrane depletion of equimolar levels of both phosphatidylethanolamine (PE) and phosphatidylcholine (PC) phospholipids (Ulus et al., 1989). Glaser and Gross (Glaser and Gross, 1995) convincingly demonstrated that membrane fusion is selectively dependent upon sufficient levels of PL, but not PE phospholipids. Specifically, sufficient levels of PL containing a polyunsaturated fatty acid at the sn-2 position (arachidonic acid, AA) but not PL containing a monounsaturated fatty acid (oleic acid, OA) or phosphatidylethanolamine (PE) containing either AA or OA at the sn-2 position was an obligate requirement for membrane fusion activity. Han et al. (Han et al., 2001) performed a comprehensive analysis of PE and PL phospholipids in both gray and white matter of different brain regions, including temporal cortex, of persons with varying degree of cognitive impairment. White matter demonstrated a preponderant loss of PL containing a monounsaturated fatty acid at sn-2 and gray matter demonstrated a preponderant loss of PL containing a polyunsaturated fatty acid at sn-2 (AA and docosahexaenoic acid, DHA).

The temporal cortex was selected as a representative brain region of interest due the previous detailed characterization of PL and PE species by Han et al. (Han et al., 2001); the observation that this brain region exhibits early loss of synaptic density (Scheff et al., 2011); the association between amyloid and tau accumulation in this region and early dysfunction in AD (Halawa et al., 2019); and its early association with aging and cognitive decline (Braak and Braak, 1991; Bancher et al., 1993; Braak et al., 1996; Braak and Braak, 1997). A representative and matched series of PE and PL species containing the same carbon number at sn-1 (stearic, 18:0) and identical sn-2 fatty acids [OA (18:1), linoleic, LA (18:2); AA (20:4); DHA (22:6)] were chosen such that differential associations between cognition and pathology and sn-1 composition (plasmenyl vs. phosphatidyl) and sn-2 composition [side chain length (18, 20, 22) and unsaturation (1,2,4,6)] could be evaluated. Accordingly, the only difference between the two series of phospholipids investigated is that the sn-1 stearic (18:0) moiety in the PL species is connected to the glycerol backbone *via* a fatty alcohol vinyl ether bond and sn-1 stearic (18:0) moiety in the PE species is connected to the glycerol backbone *via* a fatty acid ester bond. The chemical compositions and structures of the two phospholipid series are identical in all other aspects.

## Materials and methods

### Brain samples

One hundred inferior temporal cortex brain samples were obtained from the Rush University Memory and Aging Project (MAP). Rush MAP is a community-based study that enrolls older persons without known dementia who agreed to annual clinical and cognitive evaluations and to donate their brains after death (Bennett et al., 2012; Yu et al., 2017). Brain samples were fractioned and immediately frozen and stored at −80° until analysis (average post-mortem interval of approximately 8 h). All participant samples were processed under the same protocol and no significant differences in sample collection times were observed (Table 1). At the last clinical visit prior to the death and subsequent autopsies of the donors, 38 had no cognitive impairment (NCI), 44 had mild cognitive impairment (MCI) and 38 had dementia of the Alzheimer’s type (AD). Genders of the study participants were equally distributed (52 females and 48 males) and the average age was 88.5 ± 5.8 years old. A uniform structured clinical evaluation was performed to document the level of cognition and the presence of AD, MCI, and other causes of cognitive impairment. Neuropsychological indices of cognition were summarized as a global measure of cognition (Gcog) based on the average z-score of 19 tests in each study, using the means and standard deviations from the baseline visit. Participant characteristics and the number of people in each clinical category is given in Table 1.

**TABLE 1:** Demographic, clinical and biochemical summary of the study cohort.

Variable	NCI^a^	MCI	AD		Cognitively normal	Non-cognitively normal		Non-demented	Demented	*p*-value
	Mean (SD)	Mean (SD)	Mean (SD)	*p*-value	Mean (SD)	Mean (SD)	*p*-value	Mean (SD)	Mean (SD)	
Gender
Male (*n*)	18	11	19	9.5e−1	18	30	9.2e−1	29	19	7.5e−1
Female (*n*)	20	13	19		20	32		33	19
Age (SD)	86.7 (6.16)	88.6 (5.66)	90.2 (5.01)	2.6e−2*	86.7 (6.16)	89.6 (5.29)	1.4e−2*	87.4 (5.99)	90.2 (5.01)	1.7e−2*
Education (SD)	14.2 (2.79)	14.7 (2.16)	14.8 (3.20)	1.5Ee−1	14.2 (2.79)	14.7 (2.82)	3.4e−2*	14.4 (2.55)	14.8 (3.19)	4.7e−1
Post-mortem Interval (min)	369.4 (136.3)	492.5 (320.2)	394.2 (272.0)	5.3e−1	369.4 (136.3)	431.3 (292.4)	2.3e−1	416.6 (230.7)	394.2 (272.0)	6.6e−1
MMSE	28.4 (1.59)	25.6 (2.99)	12.0 (8.05)	<1.0e−5*	28.4 (1.59)	17.4 (9.32)	<1.0e−5*	27.3 (2.61)	12.0 (8.04)	<1.0e−5*
Gcog	0.213 (0.421)	−0.436 (0.33)	−1.91 (1.01)	<1.0e−5*	0.213 (0.42)	−1.33 (1.08)	<1.0e−5	−0.04 (0.50)	−1.91 (1.01)	<1.0e−*
Braak	*n*	*n*	*n*	*p*-value	*n*	*n*	*p*-value	*n*	*n*	*p*-value
I/II	13	6	2	<1.0e−3*	13	8	<1.0e−3*	19	2	<1.0e−3*
III	14	7	5		14	12		21	5	
IV	11	5	12		11	17		16	12	
V/VI	0	6	19		0	25		6	19	
Cerad	*n*	*n*	*n*	*p*-value	*n*	*n*	*p*-value	*n*	*n*	*p*-value
1	6	4	25	<1.0e−3*	6	29	8.0e−3*	10	25	<1.0e−3*
2	12	7	9		12	16		19	9	
3	5	5	2		5	7		10	2	
4	15	8	2		15	10		23	2	
Tangles	3.13 (3.92)	7.03 (8.39)	13.8 (13.7)	<1.0e−5*	4.45 (3.92)	11.1 (12.3)	2.0e−4*	4.64 (6.29)	13.8 (13.7)	<1.0e−5*
Amyloid	2.62 (2.95)	3.52 (4.33)	5.03 (3.20)	1.1e−2*	2.62 (2.95)	4.44 (3.72)	1.3e−2*	2.97 (3.55)	5.02 (3.20)	4.5e−3*
PE 18:0/18:1	0.037 (0.085)	−0.001 (0.087)	−0.074 (0.127)	<1.0e−5*	0.04 (0.09)	−0.05 (0.12)	3.0e−4*	0.02 (0.09)	−0.07 (0.13)	<1.0e−5*
PE 18:0/18:2	0.052 (0.131)	−0.031 (0.132)	−0.089 (0.117)	<1.0e−5*	0.05 (0.13)	−0.07 (0.12)	<1.0e−5*	0.02 (0.13)	−0.09 (0.12)	1.0e−4*
PE 18:0/20:4	0.034 (0.076)	0.012 (0.084)	−0.074 (0.119)	<1.0e−5*	0.03 (0.08)	−0.04 (0.11)	6.0e−4*	0.03 (0.08)	−0.07 (0.12)	<1.0e−5*
PE 18:0/22:6	0.034 (0.070)	0.017 (0.079)	−0.072 (0.105)	<1.0e−5*	0.03 (0.07)	−0.04 (0.11)	3.0e−4*	0.03 (0.07)	−0.07 (0.11)	<1.0e−5*
PL 18:0/18:1	−0.073 (0.239)	−0.084 (0.242)	−0.078 (0.236)	9.9e−1	−0.08 (0.24)	−0.08 (0.24)	8.8e−1	−0.08 (0.24)	−0.08 (0.24)	9.8e−1
PL 18:0/18:2	0.014 (0.120)	−0.032 (0.116)	−0.039 (0..117)	1.2e−1	0.01 (0.12)	−0.04 (0.12)	4.0e−2*	−0.004 (0.13)	−0.04 (0.12)	1.5e−1
PL 18:0/20:4	0.019 (0.064)	0.009 (0.074)	−0.051 (0.114)	2.0e−3*	0.02 (0.06)	−0.03 (0.10)	1.5e−2*	0.02 (0.07)	−0.05 (0.11)	4.0e−4*
PL 18:0/22:6	0.032 (0.071)	0.004 (0.055)	−0.054 (0.088)	<1.0e−5*	0.03 (0.07)	−0.03 (0.08)	1.0e−4*	0.02 (0.07)	−0.05 (0.09)	<1.0e−5*
Flotillin	0.039 (0.015)	0.037 (0.012)	0.052 (0.020)	4.0e−4*	0.04 (0.02)	0.05 (0.02)	7.9e−2	0.04 (0.01)	0.05 (0.02)	1.0e−4*

NCI, No cognitive impairment; MCI, Mild Cognitive Impairment; AD, Alzheimer’s Disease; SD, Standard Deviation. *p < 0.05.

a Clinical and biochemical differences between clinical entities were compared using analysis of variance.

### Neuropathological measurements

Global cortical amyloid load and PHF-tangle density were assessed separately using immunohistochemical methods (Yu et al., 2017). Braak staging and CERAD scoring were based on blinded assessment by a trained neuropathologist (Yu et al., 2013). Braak Stage is a semiquantitative measure of severity of neurofibrillary tangle (NFT) pathology (range 1–6; 6 = severe neocortical involvement). CERAD score is a semiquantitative measure of neuritic plaques (range: 1–4; 1 = definite AD; 4 = No AD). Flotillin-1 (FLOT1) mRNA expression was measured by qPCR. Total messenger RNA was extracted from cells using TRIzol^™^ following the manufacturer’s instructions (ThermoFisher). qPCR was conducted using a Nanodrop 1000 (ThermoFisher). The primers used were as follows: forward: 5′-CCC​ATC​TCA​GTC​ACT​GGC​ATT-3′ and reverse: 5′-CCG​CCA​ACA​TCT​CCT​TGT​TC-3′ for FLOT1 and β-actin was used as an internal reference.

### Tissue ethanolamine phospholipid measurements

Brain samples were processed essentially as described in a previous publication (Miville-Godbout et al., 2016). Briefly, brain samples were randomized prior to processing and then homogenized and ground to a fine powder in liquid nitrogen. Tissue samples were aliquoted (1 mg), water (200 μl) was added and samples were sonicated in an ice bath. After adding 600 μl of EtOAc, samples were shaken at 2000 rpm for 15 min followed by a 10 min centrifugation at 2851 *g*. An aliquot of the upper organic layer (36 μl) was then diluted with 420 μl of a solution of 0.5% water in EtOAc containing labeled internal standards [1 μg/ml ^13^C-PL (C_37_^13^C_6_H_74_NO_7_P) and 1 μg/ml of ^13^C-PE (C_24_^13^C_19_H_74_NO_8_P)]. Samples were again shaken at 2000 rpm for 15 min followed by a 2 min centrifugation at 2851 *g* from which a 100 μl aliquot was analyzed by flow injection LC-MS/MS as described previously (Goodenowe et al., 2007; Miville-Godbout et al., 2016). Briefly, the speciation of the ethanolamine phospholipids of interest were analyzed using multiple reaction monitoring (MRM) of one parent/fragment transition for the ion pairs (Table 2) on an API4000^™^ mass spectrometer equipped with a TurboV^™^ source in the negative ionization atmospheric pressure chemical ionization mode. Negatively charged PE and PL parent ion species fragment under collision induced dissociation. The negative charge of the parent ion is retained on the sn-2 fatty acid fragment in PL species and either the sn-1 or sn-2 fatty acid fragment in PE species. Therefore the combination of the [M-H]- parent ion and the sn-1 or sn-2 [R]- ion uniquely identifies the PE or PL species of interest (Goodenowe et al., 2007). Similarly, negative ion MS/MS analysis of phosphatidylcholine species enables speciation determination (Ritchie et al., 2016). Stable isotope ratios for each analyte were calculated and used for all analyses.

**TABLE 2:** MRM transitions used for ethanolamine phospholipid.

Phosphatidylethanolamines (PE)			Ethanolamine plasmalogens (PL)		
Analyte	Molecular Formula	MRM Transition	Analyte	Molecular Formula	MRM Transition
^13^C-PE 16:0/22:6	C_24_^13^C_19_H_74_NO_8_P	781.5/327.2	^13^C-PL 16:0/22:6	C_37_^13^C_6_H_74_NO_7_P	752.5/327.2
PE 18:0/18:1	C_41_H_80_NO_8_P	744.5/283.2	PL 18:0/18:1	C_41_H_80_NO_7_P	728.5/281.2
PE 18:0/18:2	C_41_H_78_NO_8_P	742.5/283.2	PL 18:0/18:2	C_41_H_78_NO_7_P	726.5/279.2
PE 18:0/20:4	C_43_H_78_NO_8_P	766.5/283.2	PL 18:0/20:4	C_43_H_78_NO_7_P	750.6/303.2
PE 18:0/22:6	C_45_H_78_NO_8_P	790.5/283.2	PL 18:0/22:6	C_45_H_78_NO_7_P	774.5/327.2

MRM, Multiple reaction monitoring.

### Statistical analyses

In descriptive analyses, demographic and clinico-pathological parameters among diagnostic categories (NCI, MCI and AD) were compared using chi-square or Fishers’s exact tests for categorical variables and analysis of variance for continuous variables. PL and PE analytes were first mean normalized and then log transformed to reduce skewness.

For continuous outcomes such as Gcog, we first screened potential predictors by examining correlations with the outcome and then fit multiple linear regression models including variables of interest and terms to adjust for age, sex and education. For continuous predictors, associations are shown per SD of the predictor. Stata 15.0 was used to perform statistical analyses; statistical significance is *p* < 0·05 in all analyses.

## Results

### Key associations with study outcomes

First, we examined the age, education, genotype, post-mortem interval, Mini-Mental State Examination (MMSE) score, Gcog, Braak stage, CERAD score, tangle density, amyloid density, brain levels of selected PL and PE phospholipids and flotillin-1 expression differences between the diagnostic categories (Table 1). The age, MMSE score, Gcog, Braak stage, Cerad score, tangle density, amyloid density, flotillin-1 expression, each of the PE and two of the PL species (PL 18:0/20:4 and 18:0/22:6) differed significantly between the diagnostic categories.

We also examined how these variables differed between cognitively unimpaired persons (NCI) and cognitively impaired persons (MCI and AD), and between non-demented (NCI and MCI) and demented (Alzheimer’s dementia) persons. Age and education were higher (*p* < 0.05) in cognitively impaired persons compared to cognitively unimpaired persons (Table 1). Demented persons were older (*p* < 0.05) than non-demented persons. As expected, MMSE and Gcog were lower (*p* < 0.05) in cognitively impaired persons and in demented persons compared to cognitively unimpaired and non-demented persons respectively. Similarly, there were more individuals with higher Braak staging, increased tangle density, increased amyloid density and lower Cerad scoring (*p* < 0.05) in cognitively impaired and demented groups than their respective cognitively unimpaired and non-demented counterparts. Each of the PE species and PL 18:0/20:4 and PL 18:0/22:6 was significantly lower in the brain of individuals belonging to the cognitively impaired and demented categories compared to cognitively unimpaired and non-demented categories respectively. PL 18:0/18:2 brain levels were significantly lower in cognitively impaired persons compared to cognitively unimpaired persons and levels of this species was similar between non-demented and demented. Flotillin-1 gene expression was significantly higher in demented persons than non-demented persons (Table 1).

### Association of degree of unsaturation and chain length of PL and PE with cognition

We examined the association between brain levels of monounsaturated fatty acid (MUFA) and PUFA-containing (at sn-2) PE and PL with Gcog and found that higher levels of each PE species predicted higher Gcog irrespective of the level of unsaturation at the sn-2 position. However, only higher levels of highly unsaturated AA (20:4, n-6) or DHA (22:6, n-3)-containing PL was associated with higher Gcog.

**FIGURE 1:**
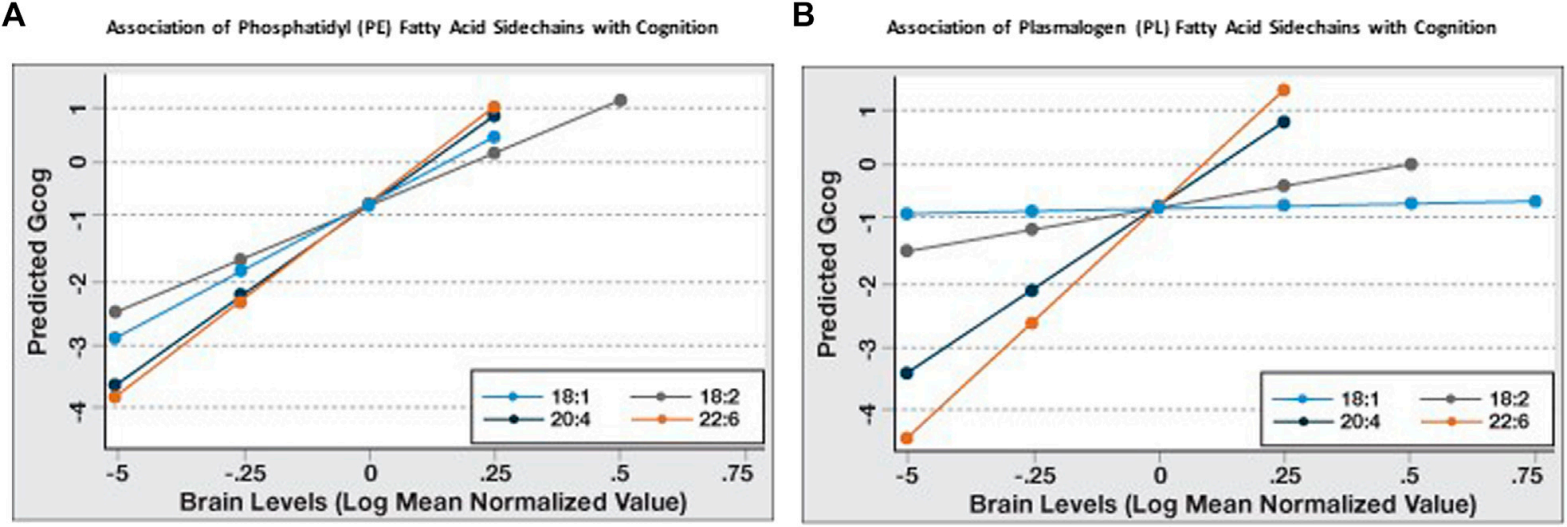
Sn-2 fatty acid sidechain-specific associations of phosphatidyl (PE) and plasmalogen ethanolamines (PL) with cognitive function (Gcog). Regression analyses were conducted with Gcog as the outcome variable and respective phospholipid species with different sn-2 fatty acid side chains as the independent variable. Four separate models for each phospholipid species, adjusted for age, sex and education, were constructed and postestimation predicted value of the outcome variable by brain levels of respective phospholipid were plotted in a single graph for comparison. **(A)** Effect of phosphatidyl ethanolamine (PE) species with different sn-2 fatty acid side chains on cognition; **(B)** Effect of plasmalogen ethanolamine (PL) species with different sn-2 fatty acid side chains on cognition.

Since it appeared that the coefficient of association of PL and PE species with Gcog was affected by the degree of unsaturation and chain length of the sn-2 sidechains, we further examined the association of the change in sn-2 chain length (18 carbon to 22 carbon) and the degree of unsaturation (one double bond to six double bonds) with cognition in each phospholipid type. We observed that for PE, the predicted Gcog values after adjusting for age, sex and education changed little based on the sn-2 side chains (Figure 1A). However, for PL, the regression slopes increased incrementally with increasing sn-2 chain length and unsaturation (Figure 1B). As expected, pathological markers were negatively associated with cognition (Table 3).

**TABLE 3:** Association of clinical and biological variables with cognition.

Variable^a^		Gcog		
	*n*	Coef^b^	*p*	Adj *R* ^2^
PE18:0/18:1	99	0.4950	6.0e−06*	0.2346
PE18:0/18:2	99	0.4882	1.7e−05*	0.2180
PE18:0/20:4	99	0.5983	4.1e−08*	0.3095
PE18:0/22:6	99	0.5989	2.4e−08*	0.3174
PL 18:0/18:1	99	0.0380	7.4e−01	0.0483
PL 18:0/18:2	99	0.1692	1.4e−01	0.0691
PL 18:0/20:4	99	0.4981	9.0e−06*	0.2283
PL 18:0/22:6	99	0.6191	9.6e−09*	0.3300
Amyloid	98	−0.3901	5.7e−04*	0.1499
Tangles	99	−0.5510	6.7e−07*	0.2685
Flotillin	94	−0.2601	3.4e−02*	0.0879

a Multiple regression analysis with phospholipids or pathological parameters as the independent variable and Gcog as the dependent variable. Gcog represents a composite measure of cognitive function assessed by a battery of cognitive tests.

b Coefficients for continuous variables expressed per Standard Deviation; Models were adjusted for age, education, and gender. *p < 0.05.

Similarly, the strength of the association (β-coefficient) increased with increasing sn-2 unsaturation and chain length in both phospholipid types and DHA-containing PL and PE had the strongest association with Gcog (Table 3). Since our objective was to examine how phospholipids modulate the association of brain pathology with cognition, we limited further statistical analyses to these two phospholipids.

### Multivariate associations of brain pathology, membrane markers and phospholipids with cognition

We then examined the association of amyloid and tangles with Gcog. As expected, both amyloid and tangle density, which are hallmarks of AD-related brain pathology, predicted lower Gcog (Base Model, Table 4). We then examined whether the expression of flotillin-1, which is a marker of lipid rafts, modulate the association of amyloid and tangles with Gcog in the Base Model. Flotillin-1 was not significantly associated with cognition when amyloid and tangles were in the model and the associations of amyloid and tangles with Gcog remained intact after the addition of flotillin-1 to the model (Model A, Table 4), indicating the independence of the association of amyloid and tangles with Gcog. We then added both PE 18:0/22:6 and PL 18:0/22:6, the phospholipids most associated with Gcog from Table 3, to the model and found that these phospholipids reduced the strength of the association of amyloid and tangles with Gcog and increased the association of flotillin-1 with Gcog (*p* = 0.053) (Model B, Table 4). The strengths of the associations (as determined by the β-coefficient) of these pathological markers with cognition weakened in relation to the base model (adjusted only for age, sex and education; Table 3) when PE and PL 18:0/22:6 were added to the model, indicating that the brain levels of these phospholipids contribute to the variability explained by these pathological markers (Model B, Table 4).

**TABLE 4:** Association of amyloid, tangles, flotillin and phospholipids with cognition.

Variable	Gcog^a^			
	Base model	Model A	Model B	Final model
PL 18:0/22:6 (Coef^b^)	Not Included	Not Included	0.5194*	0.5058***
PE 18:0/22:6 (Coef)	Not Included	Not Included	−0.0520	Not Included
Amyloid (Coef)	−0.2435*	−0.2315*	−0.1267	Not Included
Tangles (Coef)	−0.4792***	−0.4569***	−0.2815*	−0.3069 **
Flotillin (Coef)	Not Included	−0.1605	−0.2029	−0.2027*
Age (Coef)	−0.2168*	−0.1574	−0.2145	−0.2109*
Education (Coef)	0.1803	0.1501	0.0622	0.0628
Male sex (Coef)	−0.0811	−0.0700	−0.0005	−0.0053
*F*	9.18***	7.71***	9.39***	12.84***
*Adjusted R* ^2^	0.2965	0.3045	0.4219	0.4331
*N*	98	93	93	94

Independent multivariate regression models with Gcog as the outcome and phospholipids and pathological parameters as variables.

a Gcog (Global Cognition) represents a composite measure of cognitive function assessed by a battery of cognitive tests.

b Coef (Coefficient of association) for continuous variables expressed per Standard Deviation; Base Model: demographics + amyloid and tangles; Model A: Base Model + Flotillin; Base Model + Flotillin and DHA ethanolamine phospholipids; Final Model: only demographics and variables independently associated with Gcog after multivariate assessment (*p* < 0.05). *p < 0.05; **p < 0.01, ***p < 0.00001.

However, the association of PE 18:0/22:6 with Gcog observed in Table 3 disappeared under multivariate analysis that included PL 18:0/22:6 (Model B, Table 4). Therefore, to derive the final model, we removed PE 18:0/22:6 and other variables that were not associated with Gcog under multivariate conditions. Removal of these variables resulted in an increase in the variance in cognition explained by the model (Final Model, Table 4). The Final Model resulted in an improvement of the association of flotillin-1 with Gcog (*p* < 0.05). Brain amyloid levels were not associated with Gcog under multivariate analysis (Model B, Final Model, Table 4). When compared to the model without PL 18:0/22:6, inclusion of PL 18:0/22:6 in the model strengthened the association of flotillin-1 with Gcog.

### Modulation of the association between cognition and PE 18:0/22:6 by PL 18:0/22:6

**FIGURE 2:**
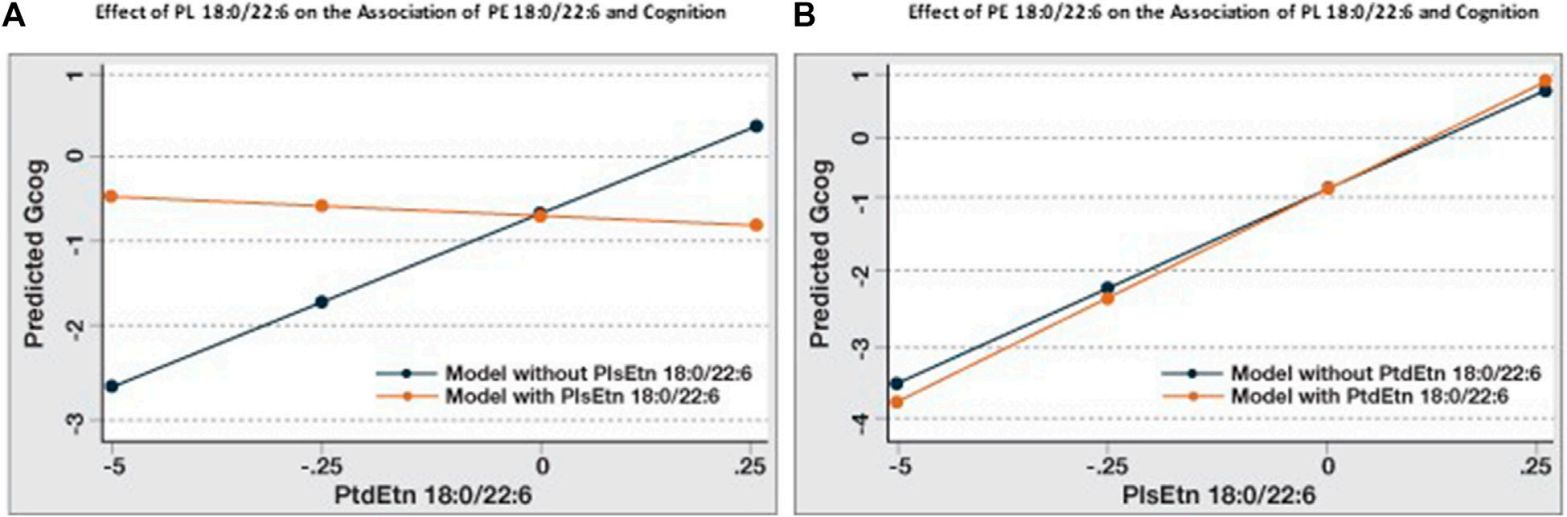
Association of PE 18:0/22:6 with cognition is modulated by PL 18:0/22:6. Regression analyses were conducted with Gcog as the outcome variable and PE 18:0/22:6 or PL 18:0/22:6 as the independent variable. Two separate models for each phospholipid, with or without the counterpart phospholipid in the model, were constructed. All models were adjusted for age, sex and education. Postestimation predicted value of the outcome variable by brain levels of the phospholipid were plotted in a single graph for comparison. **(A)** Effect of PE 18:0/22:6 on cognition with or without PL 18:0/22:6 in the model; **(B)** Effect of PL 18:0/22:6 on cognition with or without PE 18:0/22:6 in the model.

When the association between cognition and PE 18:0/22:6 in Table 3 is compared to Model B, Table 4 the addition of PL 18:0/22:6 was observed to affect this association. To investigate the relative and interactive associations of these two similar phospholipids on cognition, four predictive models were created (Figure 2). Figure 2A illustrates the association between PE 18:0/22:6 and cognition with and without PL 18:0/22:6 as a covariate. Inclusion of PL 18:0/22:6 completely neutralized the association between PE 18:0/22:6 and cognition. Figure 2B illustrates the association between PL 18:0/22:6 and cognition with and without PE 18:0/22:6 as a covariate. Inclusion of PE 18:0/22:6 had no effect on the association between PL 18:0/22:6 and cognition. These results indicate that cognition is directly associated with the level of PL 18:0/22:6 not PE 18:0/22:6.

### Modulation of the association between cognition and amyloid, tangles and flotillin by PL 18:0/22:6

**FIGURE 3:**
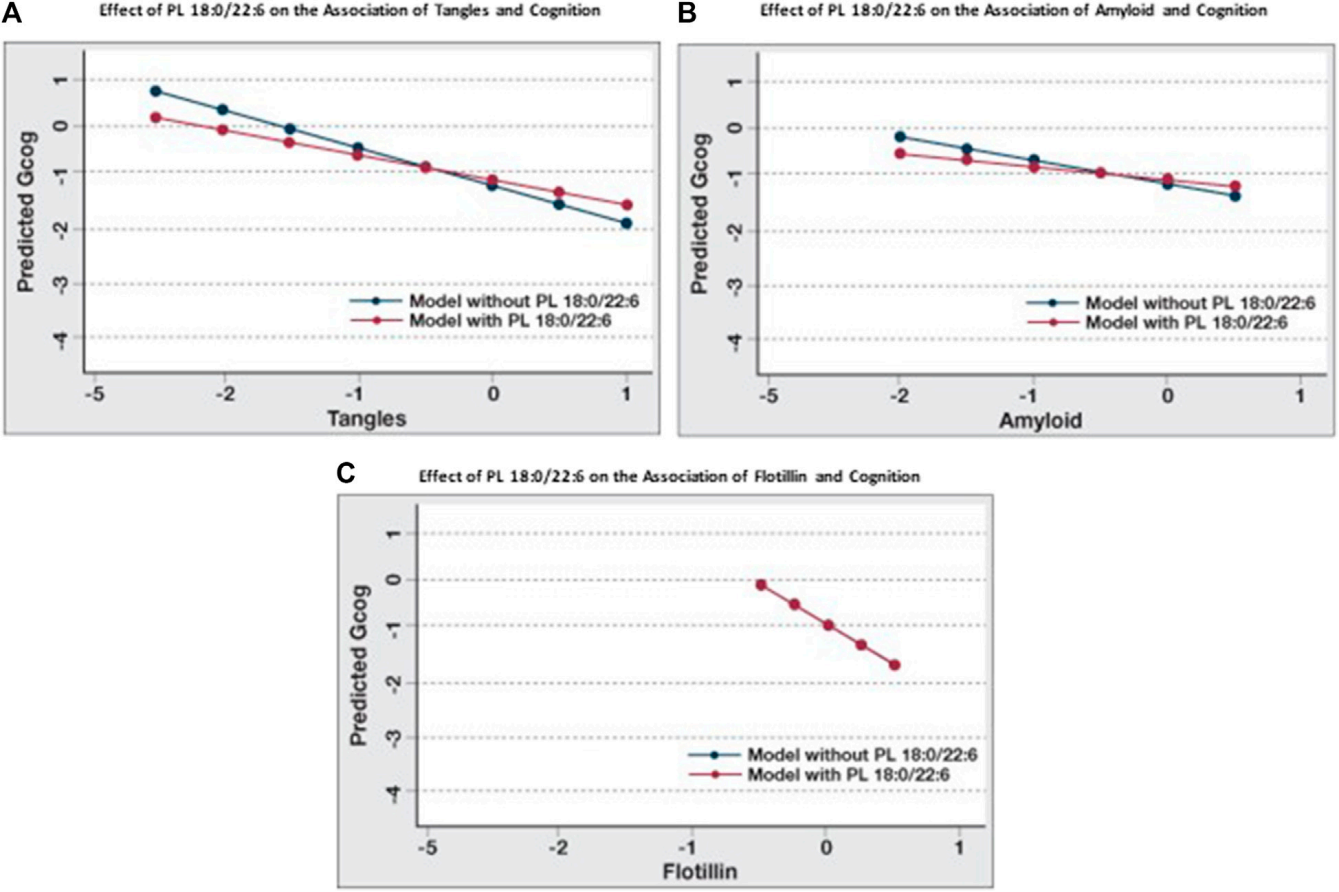
Modulation of the effects of pathological parameters on cognition by PL 18:0/22:6. Regression analyses were conducted with Gcog as the outcome variable and tangles, amyloid or flotillin respectively as the independent variable. Two separate models for each pathological parameter, with or without PL 18:0/22:6 in the model, were constructed. All models were adjusted for age, sex and education. Postestimation predicted value of the outcome variable by brain levels of the respective pathological parameter were plotted in a single graph for comparison. **(A)** Effect of tangles on cognition with or without PL 18:0/22:6 in the model; **(B)** Effect of amyloid on cognition with or without PL 18:0/22:6 in the model; **(C)** Effect of flotillin on cognition with or without PL 18:0/22:6 in the model (blue and red lines are superimposed).

When the associations between cognition and amyloid, tangles and flotillin-1 in Table 3 and Table 4 (Model A) are compared, the association between cognition and tangles is minimally affected by amyloid and flotillin (−0.55 to −0.46), but the association between amyloid and cognition is reduced almost in half (−0.39 to −0.23). When brain phospholipids are subsequently included (Model B), the association between tangles and cognition drops from −0.46 to −0.28 and the association between amyloid and cognition becomes insignificant (−0.23 to −0.13). To illustrate the effect of PL 18:0/22:6 on these associations, six predictive models were created to compare the associations between each of these pathologies and cognition with and without PL 18:0/22:6 in the model (Figure 3). Figure 3A illustrates the flattening of the association between tangles and cognition by PL 18:0/22:6. Figure 3B illustrates the flattening of the association between amyloid and cognition by PL 18:0/22:6. Amyloid is not associated with cognition when PL 18:0/22:6 is a covariate. Figure 3C illustrates that PL 18:0/22:6 has no modulatory effect on the association between flotillin-1 and cognition. What is illustrated in Figures 2, 3 which is not discernible from the data in Tables 3, 4 is the variance in cognition observed across the range of measured values of tangles, amyloid, flotillin-1 and PL 18:0/22:6. Gcog values ranged from just over 0 in persons with the lowest level of tangles to just under -1 in persons with the highest level of tangle density for a total variance in cognition of just under 2 Gcog units across the range of measured brain tangle density. The total variance in Gcog across the measured range of amyloid levels was approximately 0.5 Gcog units and the total variance of cognition across the range of flotillin-1 values was approximately 1.5 Gcog units. Whereas in Figure 2, the total variance in cognition across the range of PL 18:0/22:6 values was approximately 5 Gcog units (from −4 in persons with the lowest brain PL 18:0/22:6 levels to +1 in persons with the highest brain PL 18:0/22:6 levels).

### Association of brain pathology, flotillin and PL 18:0/22:6 with normal cognition and dementia

Multivariate regression analyses identified brain levels of PL 18:0/22:6, tangles and flotillin-1 as key predictors of cognition. To understand the nature of these associations across the full range of their measured values, we categorized the levels of these variables into low (<mean-1SD), median (mean ± 1SD) and high (>mean+1SD) and used logistic regression to determine the associations of these categories with different ends of the measurable cognition range. The terms “no cognitive impairment, NCI” and “cognitively normal, CN” are not synonymous with high cognitive function. In fact, the range of cognitive performance in individual neuropsychometric tests in persons determined, clinically, to be NCI or CN represents a distribution with an equal number of persons above normal and an equal number of persons below normal, but above cognitive impairment (Bennett et al., 2006). Likewise, there is a distribution of cognition in persons determined, clinically, to be demented ranging from mild to severe dementia, but lower than a determination of questionable or mild cognitive impairment (MCI). Therefore, the prediction of CN from non-CN (MCI + dementia) and demented from non-demented (CN + MCI) is not an equal but opposite analysis. The lack of a negative association is not the same as the presence of a positive association. We reasoned that with these analyses, we would be able to determine and compare the strength of the positive and negative associations of lower and higher levels of these variables with cognition.

The analysis results indicated that high brain PL 18:0/22:6 (>mean+1SD) predicted normal cognition and non-demented state, but low tangle, flotillin-1 or amyloid levels did not (Table 5). However, low brain PL 18:0/22:6 (<mean-1SD) and high levels of tangles and flotillin-1 (>mean+1SD) and intermediate levels of amyloid (mean ± 1SD) predicted dementia (Table 5).

**TABLE 5:** Association of low, medium and high levels of PL 18:0/22:6, amyloid, tangles and flotillin with normal cognition and Dementia.

Variable^a^	Cognitively	Non-demented vs. demented
	Abnormal vs. NCI	
	Coefficient (95% CI)	Coefficient (95% CI)
PL 18:0/22:6		
<Mean-1SD	Ref	2.73 (1.00 to 4.46)**
Mean ± 1SD	0.56 (−0.82 to 1.95)	1.52 (0.06 to 2.99)
>Mean+1SD	1.67 (0.27 to 3.06)*	Ref
Tangles	
<Mean-1SD	17.1 (−4108.5 to 4142.6)	Ref
Mean ± 1SD	16.8 (−4108.8 to 4142.4)	0.62 (−1.38 to 2.61)
>Mean+1SD	Ref	4.85 (1.44 to 8.26)**
Flotillin	
<Mean-1SD	0.74 (−0.93 to 2.41)	Ref
Mean ± 1SD	−0.13 (−1.38 to 1.12)	1.65 (−0.35 to 3.65)
>Mean+1SD	Ref	2.27 (0.012 to 4.52)*
Amyloid	
<Mean-1SD	0.62 (−1.41 to 2.66)	Ref
Mean ± 1SD	0.43 (−1.51 to 2.37)	2.87 (0.57 to 5.17)*
>Mean+1SD	Ref	1.53 (−1.15 to 4.20)

a Logistic regression analysis to determine the effects of low, medium and high levels of phospholipids and pathological parameters on cognitively normal state (NCI) and dementia diagnosis. Dummy variables were created from continuous variables of these parameters to derive categories representing low, medium and high levels. Each model was adjusted for age, sex and education in addition to PL 18:0/22:6, tangles, flotillin as continuous variables, when the corresponding categorical variable is not included in the model. NCI, No cognitive impairment. *p < 0.05; **p < 0.01.

## Discussion

In our previous studies, we demonstrated that serum plasmalogen levels were associated with cognitive outcomes (Goodenowe et al., 2007; Wood et al., 2010; Goodenowe and Senanayake, 2019). We have also demonstrated neuroprotective effects of exogenous supplementation of plasmalogen precursors in animal models (Wood et al., 2011a; Gregoire et al., 2015; Miville-Godbout et al., 2016) and in a preliminary 4-month, escalating dose, open label study of a targeted equimolar formulation of PL 16:0/22:6 and PL 18:0/22:6 plasmalogen precursors in 22 cognitively impaired persons, blood levels of PL 16:0/22:6 and PL 18:0/22:6 were elevated by approximately 70% from baseline and this increase was associated with decreased malondialdehyde levels and increased catalase enzymatic capacity. Improved cognition and mobility were also reported (Goodenowe et al., 2022). Low brain and circulating serum plasmalogen levels in neurodegenerative diseases such as Alzheimer’s disease have been observed by multiple independent researchers and these deficiencies are thought to contribute to defects in cellular membrane function (Ginsberg et al., 1995; Farooqui et al., 1997; Han et al., 2001; Dorninger et al., 2015). In this study, we sought to examine a specific class of brain membrane lipids (ethanolamine phospholipids) based upon their known high abundance in neuronal membranes (Han, 2005), diverse sn-1 and sn-2 speciation (Han et al., 2001), and their direct physiological role in membrane fusion/neurotransmitter release (Glaser and Gross, 1995). Similarly, we selected the temporal cortex, a brain region known to exhibit pathological (Braak and Braak, 1997; Scheff et al., 2011), morphological (Braak and Braak, 1991) and functional (Halawa et al., 2019) changes at the earliest clinically detectable stages of cognitive impairment and dementia as a relevant brain region of interest.

The major findings of this study are: *1*) The association between brain levels of PL and cognition was observed to be dependent on the chain-length and the degree of unsaturation of the sn-2 fatty acid side chain. *2*) The associations between PE species and cognition were observed to be indirect and mediated *via* their association with PL 18:0/22:6. *3*) High levels (>mean+1SD) of PL 18:0/22:6 predicted normal cognition and low levels (<mean-1SD) predicted reduced cognition and dementia. In contrast, a high density of neurofibrillary tangles or high flotillin-1 expression was observed to be predictive of reduced cognition, but low levels were not predictive of normal cognition. *4*) PL 18:0/22:6 modulated the associations between cognition and PE 18:0/22:6, tangles and amyloid. *5*) Brain amyloid density was not predictive of cognition after correcting for tangle density, PL 18:0/22:6 levels, and flotillin-1 expression.

A higher age is a primary epidemiological variable associated with lower cognition and our data confirm this association (Table 1). As expected, cognitive status (Gcog), neurofibrillary tangle density, amyloid plaque density, Braak Stage (V/VI), and CERAD score significantly differed between the diagnostic subgroups (Table 1). Each of the PE species and the two PUFA-containing PL species (18:0/20:4 and 18:0/22:6) significantly differed between the diagnostic categories indicating that a global loss in brain ethanolamine phospholipid species is associated with reduced cognition. Flotillin-1 expression was significantly higher in demented individuals, which is consistent with previously published results (Girardot et al., 2003).

We observed that more double bonds (6>4>2>1) and higher carbon chain length (22>20>18) in the sn-2 fatty acid strengthened the association of PL species with cognition but had a minimal effect on the association between PE species levels and cognition. Neurotransmission involves both biophysical and biochemical processes. Prior to a putative neurotransmission event, neurotransmitter molecules are stored in presynaptic membrane-bound vesicles which isolate them from the intracellular metabolism of the presynaptic neuron. Upon receipt of a depolarizing action potential, the vesicles migrate to the outer presynaptic membrane where they fuse with and release the neurotransmitters into the extracellular synaptic cleft where they subsequently interact with protein receptors on the postsynaptic membrane of the adjacent neuron. Sufficient membrane levels of PUFA PL are required for this membrane fusion process to occur (Lohner, 1996). Efficient fusion of cellular membranes is required for effective neurotransmission (Yang et al., 2017). Membrane fusion efficiency was observed to be dependent on the amount of PL in the membrane as well as the structural composition of the PL. A longer chain length and higher degree of unsaturation of the sn-2 PL sidechain incrementally increased membrane fusion events. A direct comparison of PL and PE species of identical side chain compositions further illustrated the dependence of membrane fusion on membrane PL levels (Glaser and Gross, 1995). This is a plausible biophysical mechanism for the differential trends in the associations between PL and PE sn-2 sidechains with cognition observed in this study. Although brain plasmalogens have been shown to be lower in brain regions with AD-related pathology and the association of low serum PL levels with brain pathology have been demonstrated by us and others (Ginsberg et al., 1995; Farooqui et al., 1997; Han et al., 2001; Dorninger et al., 2015), to our knowledge this is the first direct demonstration of the compositional specificity of the PL association with cognition. Brain levels of PL with sn-2 fatty acids with longer chain length and a higher degree of unsaturation were associated with cognition whereas PL with sn-2 fatty acids with shorter chain length and a lower degree of unsaturation were not.

We examined whether the levels of PL 18:0/22:6 in the brain altered the associations between amyloid and tangles, the hallmarks of AD pathology, with cognition. Our data indicate that higher brain PL 18:0/22:6 levels decrease the strength of the association (β-coefficient) between amyloid and tangles with cognition, indicating that the association between brain pathology and cognition is affected by the level of PL 18:0/22:6 levels in the brain. These observations are consistent with the findings that amyloid and tangles do not correlate directly with cognition nor do they explain the variance of cognition in AD (Negash et al., 2011). Inclusion of PL 18:0/22:6 in the model, along with amyloid and tangles, increased the explained variance when cognition was the outcome of interest. Furthermore, when the variance in cognitive status across the full measured ranges of amyloid plaque density, neurofibrillary tangle density, flotillin-1 expression and PL 18:0/22:6 in temporal cortex were compared (Figures 1, 2) the cognitive status associated with highest versus lowest levels of these biomarkers was observed to be dramatically different. The variance in cognition across the range of amyloid density was about 0.5 Gcog units, and about 1.5 for tangles and flotillin-1, but was about 5 units for PL 18:0/22:6.

The association of PL 18:0/20:6 with cognition remained unchanged when adjusted for brain pathology (amyloid and tangles) and the increased explained variance in this model indicates that the amount of PL 18:0/20:6 in the brain is an independent contributor to cognition. Similarly, when adjusted for PL 18:0/22:6 levels, the association of PE 18:0/22:6 with cognition disappeared, while adjustment for PE 18:0/22:6 levels did not change the association of PL 18:0/22:6 with cognition. These findings suggest that PL 18:0/22:6 is an independent predictor of cognition and the association of PE 18:0/22:6 with cognition is spurious, and probably reflects the homeostatic adjustment of PE levels due to PL deficiency (Dorninger et al., 2015). Association of higher PL 18:0/22:6 levels (>mean+1SD) with normal cognition and the association of lower levels (<mean-1SD) with dementia is suggestive of protective effects at higher levels and deleterious effects at lower brain levels.

Flotillin is a marker of lipid rafts in the brain (Dermine et al., 2001). Lipid rafts are cholesterol and sphingolipid-enriched areas in the membrane that help sequester certain proteins. Lipid rafts are known to be associated with gamma-secretase that generate amyloid β-protein (Aβ) and thought to promote the interaction of the amyloid precursor protein (APP) with gamma-secretase (Matsumura et al., 2014). The apparent enhancement of the negative association of flotillin with cognition by PL 18:0/22:6 probably indicate modification of membrane structure by PL 18:0/22:6. In fact, it has been shown that increasing membrane DHA-PL levels dose dependently increased soluble APPα and decreased gamma-secretase expression (Wood et al., 2011a).

Recent research findings suggest that the onset of AD neuropathological changes occur years prior to the appearance of clinical symptoms (Sperling et al., 2014). Exploring biochemical changes associated with early pathophysiological events is crucial to finding modifiable risk factors that can be used for primary and secondary prevention (Sperling et al., 2011). PL deficiency in the brain is one such biochemical change reported to be associated with brain pathology and cognition (Ginsberg et al., 1995; Han et al., 2001; Goodenowe et al., 2007; Wood et al., 2010; Goodenowe and Senanayake, 2019). We recently reported that higher levels of serum plasmalogens weaken or nullify the associations between age and APOE e4 allele status and AD and cognition (Goodenowe and Senanayake, 2019) and that increasing circulating DHA plasmalogen levels using a plasmalogen precursor had positive effects on cognition and mobility (Goodenowe et al., 2022).

Glycerophospholipids containing a vinyl ether bond at sn-1 are called plasmalogens. They are synthetized *via* a single non-redundant biochemical pathway in intracellular organelles called peroxisomes (Brites et al., 2004). A decline in peroxisomal function due to aging leads to low PL levels in the membranes and lipoproteins in the brain causing defective membrane function (Farooqui et al., 1997) and aberrations in brain cholesterol metabolism (Goodenowe and Senanayake, 2019). These two factors lead to suboptimal cholinergic neuron function (Blusztajn and Wurtman, 1983) and atrophy and defects in amyloid clearance respectively (Sun et al., 2015), presumably leading to brain pathology characteristic of AD. PL predominates in the body over choline plasmalogens (Farooqui and Horrocks, 2001) and they play a significant role in maintaining optimal brain function (Rouser and Yamamoto, 1968; Farooqui et al., 2008). These phospholipids are associated with cellular membranes in the brain and are involved in multiple brain functions including cholesterol metabolism, synaptic function, and APP processing (Farooqui and Horrocks, 2001; Brites et al., 2004; Rothhaar et al., 2012). Alteration of membrane function is hypothesized to be mechanistically linked to the pathology and clinical symptoms observed in AD (Glaser and Gross, 1995; Askarova et al., 2011).

The study has several limitations. First it was designed to focus on a single class of lipids in a single brain region to investigate specific membrane structural changes with pathology and cognition. Extrapolation of the results to other lipid classes or brain regions or the contributions of other brain regions or other pathologies or other phospholipids to cognition was not investigated. Upstream (impaired peroxisomal function) or downstream (high levels of inflammation/oxidative stress) causal factors that may simultaneously influence PL levels and cognition was not investigated. Future studies are needed to further explore and define the roles of additional contributing factors such as these in the outcomes observed and reported in this study.

This is the first reported evidence of a potential modifiable risk factor that can modulate the effects of brain pathology on cognition possibly through membrane alterations. This report also demonstrates the spurious association of DHA-phosphatide with cognition and unequivocally demonstrates the significance of DHA-plasmalogens on cognition. Increasing DHA-plasmalogen levels in the brain might have the potential to modify the neurodegenerative pathways responsible for cognitive deficits.

## Data Availability

The raw data supporting the conclusions of this article will be made available by the authors, without undue reservation.
